# Progress in Genito-Urinary Surgery

**Published:** 1902-08-16

**Authors:** 


### PROGRESS IN GENITO-URINARY SURGERY.

(*Concluded from page* 329.)

**Nephrotomy and Nephro-lithotomy.**—Howard A. Kelly[5] has considered the technique of these operations on the basis of the anatomy of the kidney. The X-rays, he remarks, can with practical certainty be relied upon to show the presence or absence of stone in the kidney. The author's method of examining for stone by passing a bougie tipped with highly polished but impressible wax up the ureter into the kidney remains of value in cases of ureteral calculi, where it may give information as to presence and exact position of a calculus. The ureteral catheter is of use in nephro-lithotomy, firstly for thoroughly irrigating the kidney, and secondly for distending the kidney which has been exposed by the lumbar incision, causing the organ to swell up and make prominent certain surface landmarks which serve as a guide for a correct incision into the organ. In this way, by grasping the kidney and alternately distending and relaxing it, the exact positions of the calices can be detected. Also the kidney can be cut with less injury to the cortex in the distended state. The question of maximum importance is that as to the best position for making the incision through the cortex into the calices and pelvis of the kidney. Mr. Broedel has shown that in most kidneys there are two arterial supply systems separated by the renal pelvis,—a major system, carrying three-fourths of the blood, for the anterior part of the organ, and a posterior system, carrying one-fourth of the arterial blood supply. The surgeon should endeavour to make his incision in the line which divides these separated systems. If he strikes the major vessels of either system the hæmorrhage may be frightful and even fatal. There are landmarks on the surface of the kidney which show where the relatively non-vascular zone is situated. The surface of the kidney, if examined carefully, will be found divided into irregular areas corresponding to the bases of the pyramids and of about the size of the thumb end. These areas are bounded by lighter-coloured lines, often slightly depressed, and corresponding to the columns of Bertini which form the framework supporting the vessels. The white lines come together in a longitudinal white line on the anterior surface which Kelly proposes to call Broedel's white line. If the white lines are indistinct, stellate veins may show the position they usually occupy. The kidney should be incised along a line parallel to Broedel's line, but 1 cm. posterior to it, and parallel to the posterior surface of the kidney, *i.e.* through the lateral portion of the posterior pyramids, leaving about three-fifths of the kidney anterior, and two-fifths posterior. About two-thirds of all kidneys are constructed on the above lines. The rule should therefore be for the incision to be made on that side of the kidney where the palpating fingers, placed on the pelvis, feel the lesser number of vessels. Kelly would only incise the pelvis of the kidney for small stones, and when that part can be exposed, incised and sutured without much difficulty. An improved method of suturing the incision in the kidney is described in the paper.

[5] Brit. Med. Jour., Feb. 1, 1902, p. 257.

